# Supplementary material for: Effects of a Nanonetwork-Structured Soil Conditioner on Microbial Community Structure
Source: Biology (Basel). 2023 Apr 28;12(5):668. doi: 10.3390/biology12050668 (PMC10215273; doi:10.3390/biology12050668)
Supplement: Supplementary file 1 [file biology-12-00668-s001.zip › biology-2315233-supplementary.pdf]

Supplementary Materials for

Effects of a Nanonetwork-Structured Soil Conditioner on Microbial Community Structure

Jingjing Cao [1,2,†,*], Pan Zhao [1,2,3,†,*], Dongfang Wang [4], Yonglong Zhao [1,2], Zhiqin Wang [1,2], Naiqin Zhong [1,2,3,*]

1 State Key Laboratory of Plant Genomics, Institute of Microbiology, Chinese Academy of Sciences, Beijing 100101, China; caojingjing@im.ac.cn (J.C.); ylzhao97101@163.com (Y.Z.); wangzq1210@163.com (Z.W.);
2 Engineering Laboratory for Advanced Microbial Technology of Agriculture, Chinese Academy of Sciences, Beijing 100101, China
3 The Enterprise Key Laboratory of Advanced Technology for Potato Fertilizer and Pesticide, Hulunbuir 021000, China
4 College of Environmental Science and Engineering, Donghua University, Shanghai 201620, China; dfwang@dhu.edu.cn (D. W.)
* Correspondence: zhaop@im.ac.cn (P.Z.); nqzhong@im.ac.cn (N.Z.)
† These authors contributed equally to this work.

This file includes:

Figure S1 to S6
Tables S1

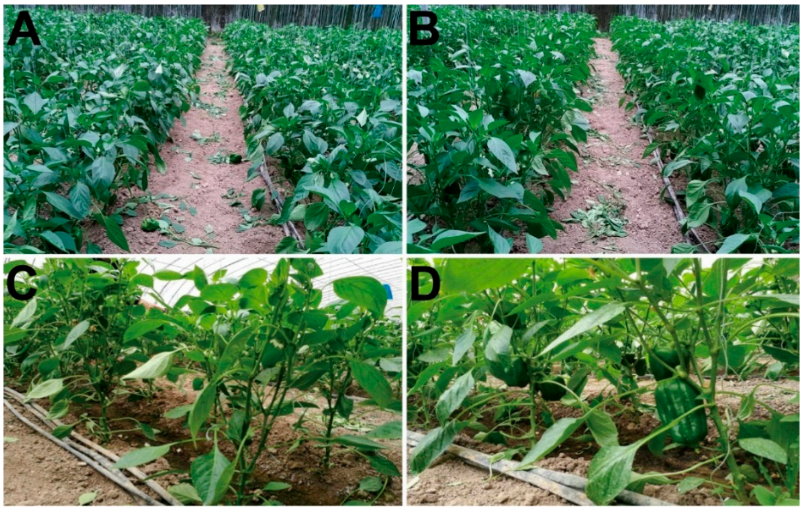

Figure S1. Photographs of soil conditioner on pepper. (A and C) Plants growing in CK; (B and D) plants growing in SC3.

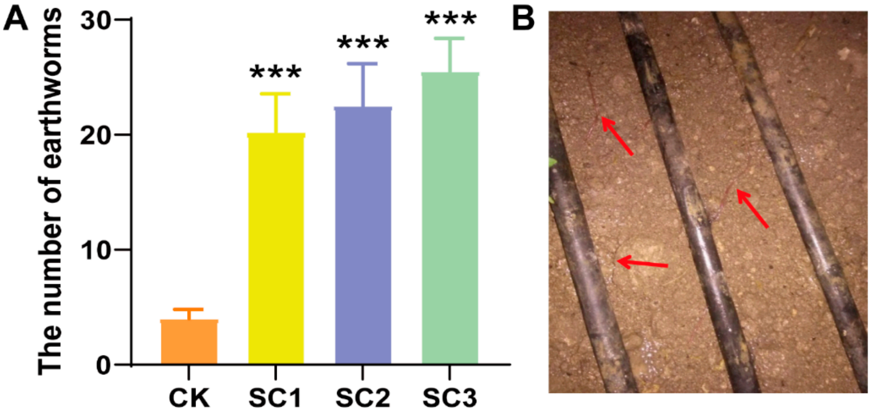

Figure S2. The number of earthworms. (A) The number of earthworms in soil treated with different amounts of SC. (B) Digital photograph of earthworms in soil with SC3 (400 kg/acre) treatment (*** $p < 0.001$).

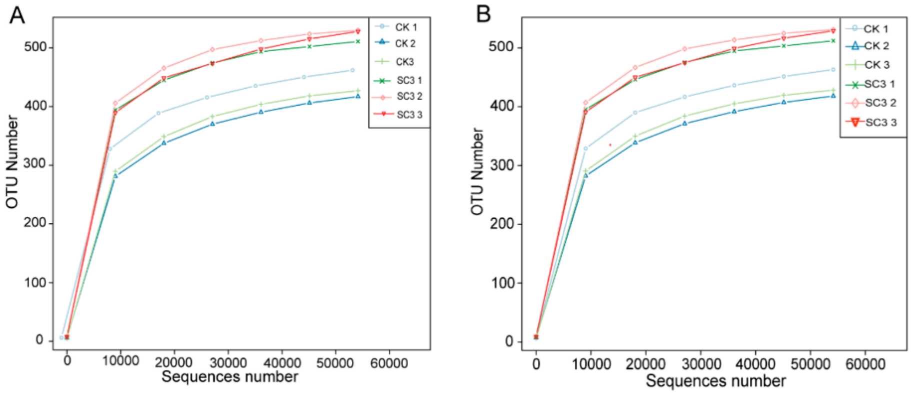

Figure S3. The rarefaction curve of samples. (A) 16S rRNA; (B) ITS. Rarefaction curves of OTUs clustered for a dissimilarity of 3%.

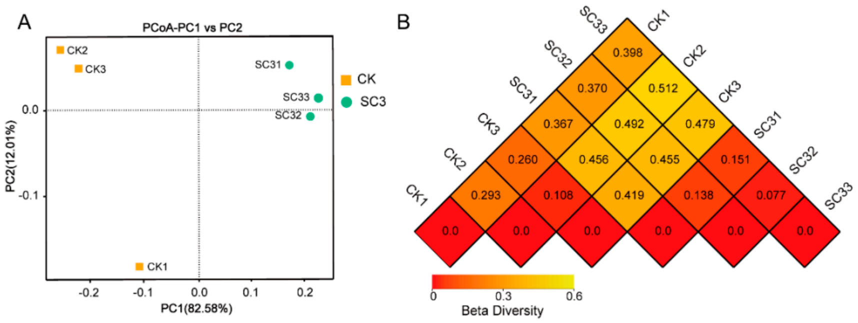

Figure S4. Beta-diversity analysis showing the relatedness of bacterial communities in different samples. (A)The principle co-ordinate analysis (PCoA). (B) The heatmap of beta-diversity index. The number in the square represents the diversity between the two samples. The bigger the number, the more difference in beta-diversity.

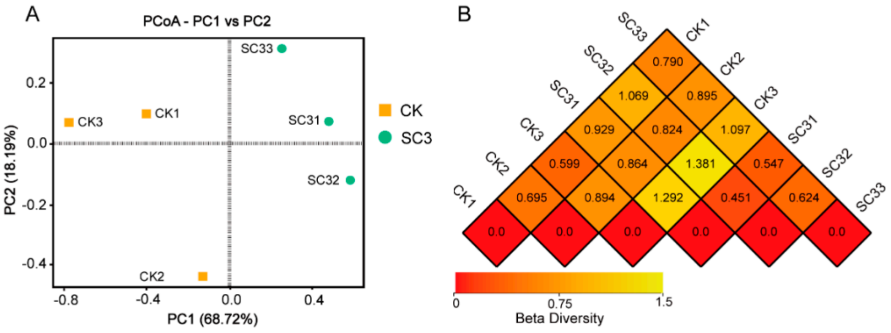

Figure S5. Beta-diversity analysis showing the relatedness of fungal communities in different samples. (A)The principle co-ordinate analysis (PCoA). (B) The heatmap of beta-diversity index. The number in the square represents the diversity between the two samples. The bigger the number, the more difference in beta-diversity.

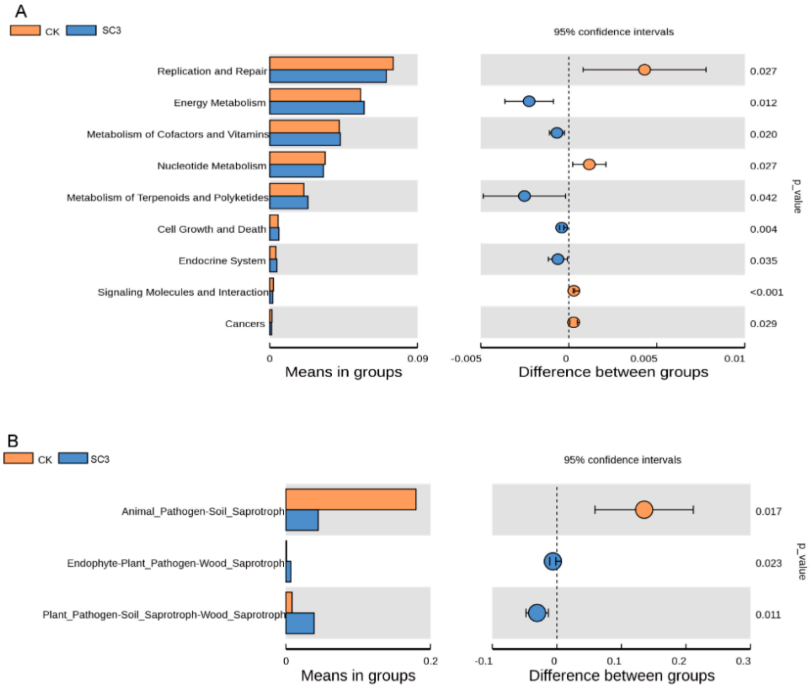

Figure S6. Difference analysis via *t*-test based on the results of functional annotation. (A) Functional analyses for bacterial communities via PICRUSt; (B) Functional analyses for fungal communities via FUNGuild.

Table S1. Sequencing statistic

	Sample_name	Clean_Reads	Base (nt)	AvgLen (nt)	Q20	GC%	Effective%
16S rRNA	CK1	80123	33149469	413	81.59	55.6	94.12
	CK2	80346	33453661	416	82.02	54.8	91.73
	CK3	92494	38467922	415	80.97	55.06	97.16
	SC31	86348	35728107	413	76.89	56.9	96.71
	SC32	90690	37493233	413	79.11	57.32	91.08
	SC33	80149	33127959	413	78.8	57.22	94.06
ITS1-5F	CK1	80130	18795920	234	67.91	44.45	96.84
	CK2	80193	18332893	228	78.76	46.2	96.07
	CK3	80215	19170951	238	67.63	47.15	97.27
	SC31	80161	17689391	220	72.49	45.95	98
	SC32	80138	18029856	224	81.57	46.62	98.13
	SC33	80081	18838169	235	85.11	45.99	96.25
