# Supplementary figures and images for: Effects of a Nanonetwork-Structured Soil Conditioner on Microbial Community Structure
Source: Biology (Basel). 2023 Apr 28;12(5):668. doi: 10.3390/biology12050668 (PMC10215273; doi:10.3390/biology12050668)

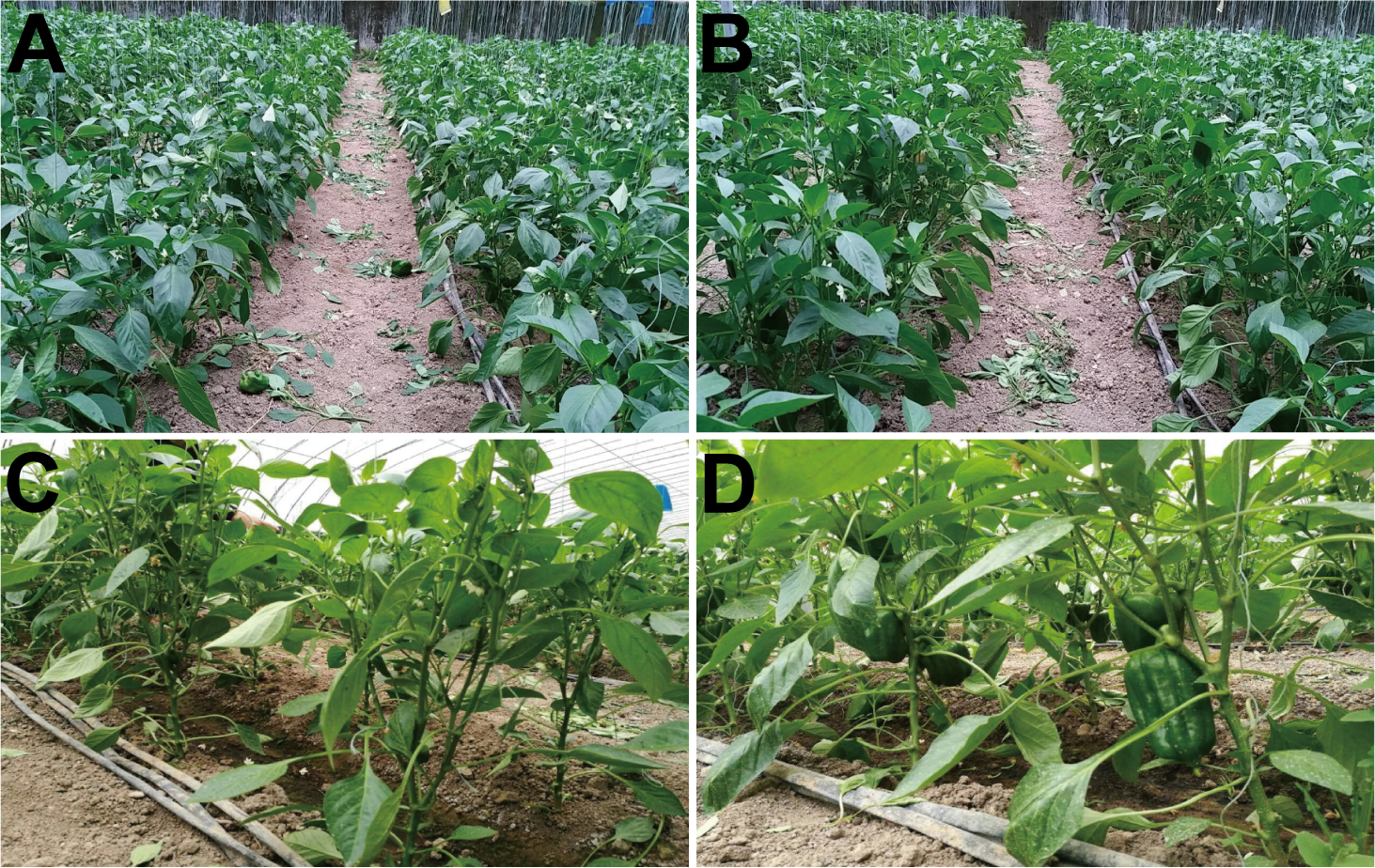

Supplement: Supplementary file 1 [file biology-12-00668-s001.zip › Figure S1.tif]

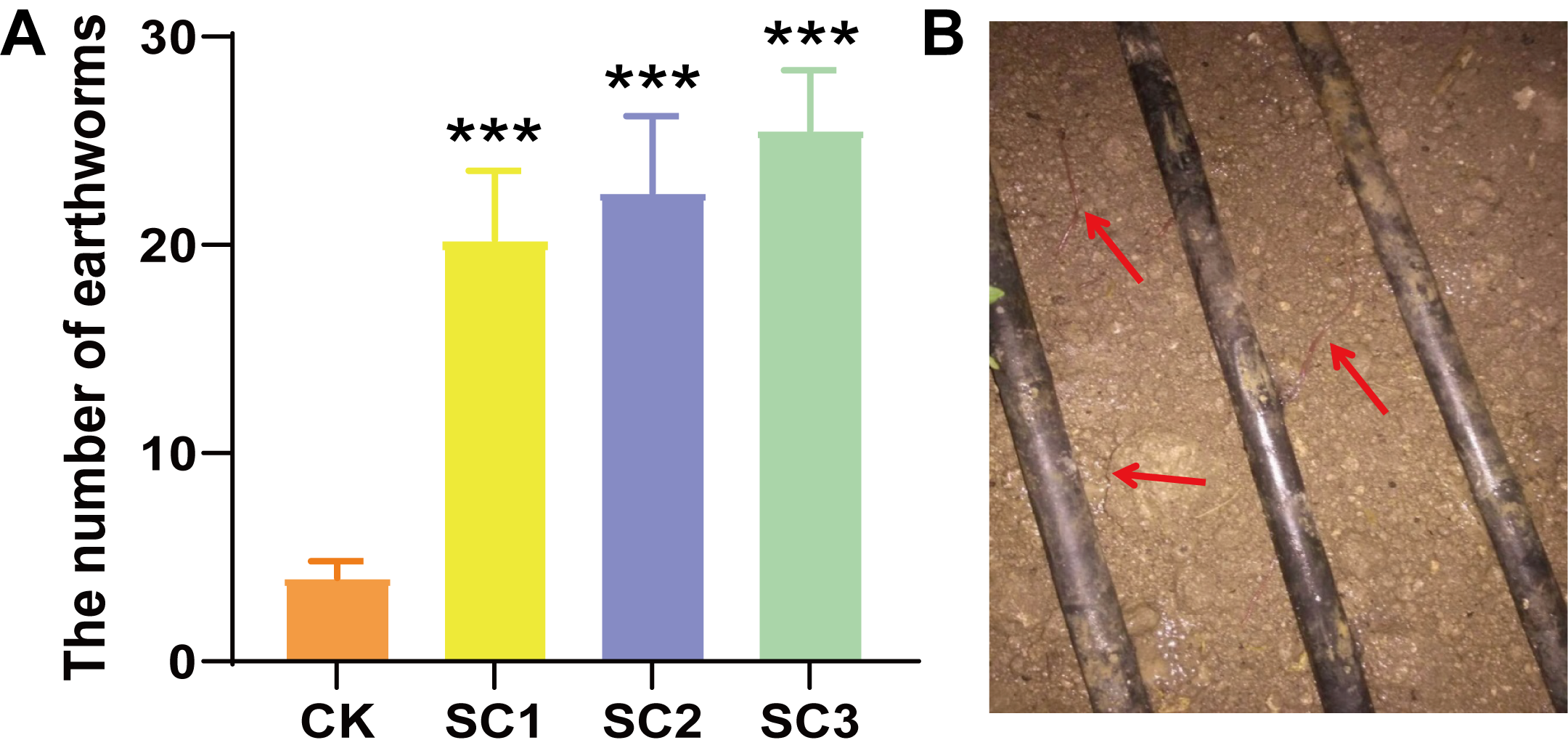

Supplement: Supplementary file 1 [file biology-12-00668-s001.zip › figure S2.tif]

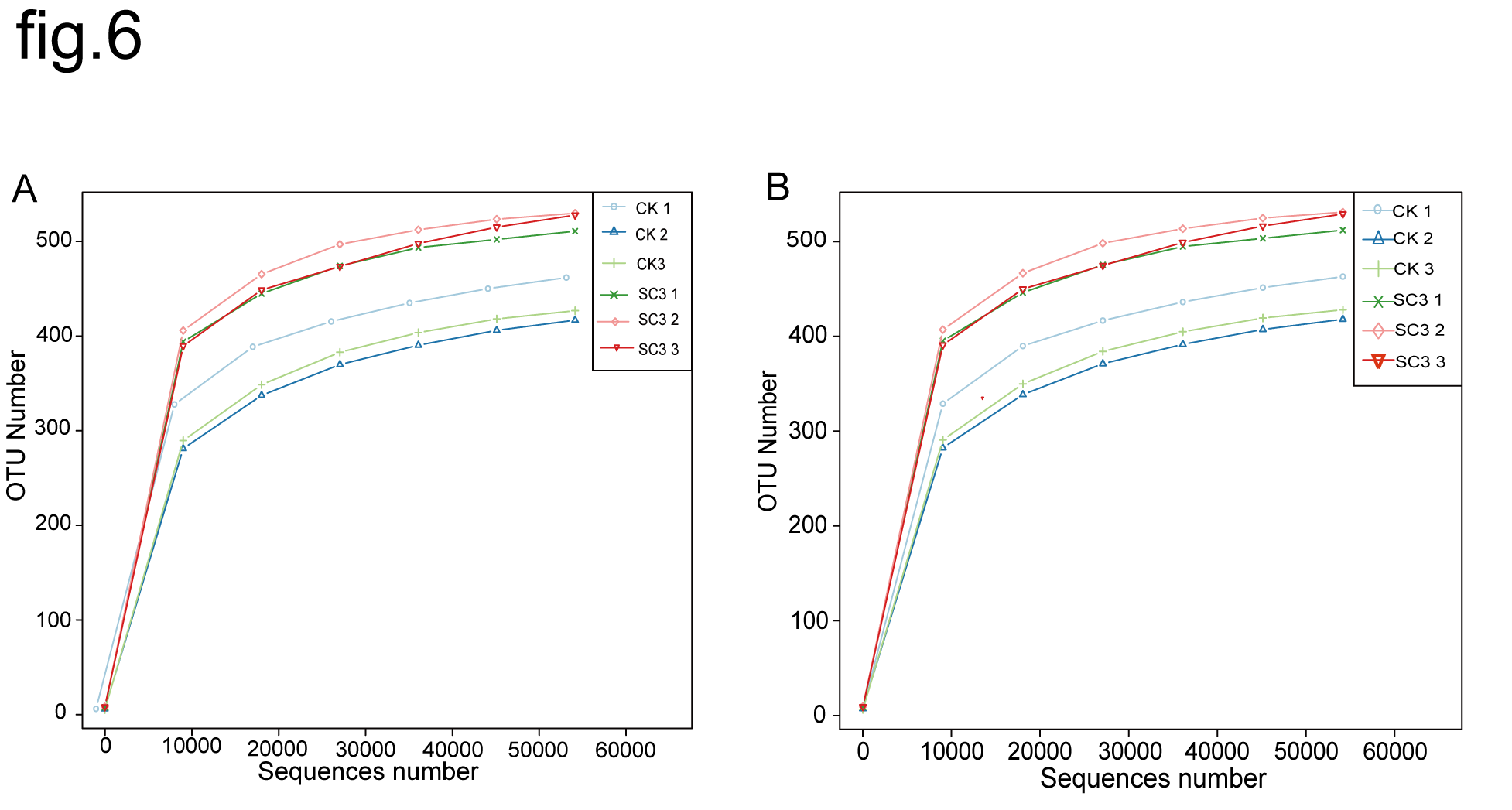

Supplement: Supplementary file 1 [file biology-12-00668-s001.zip › figure S3 .tif]

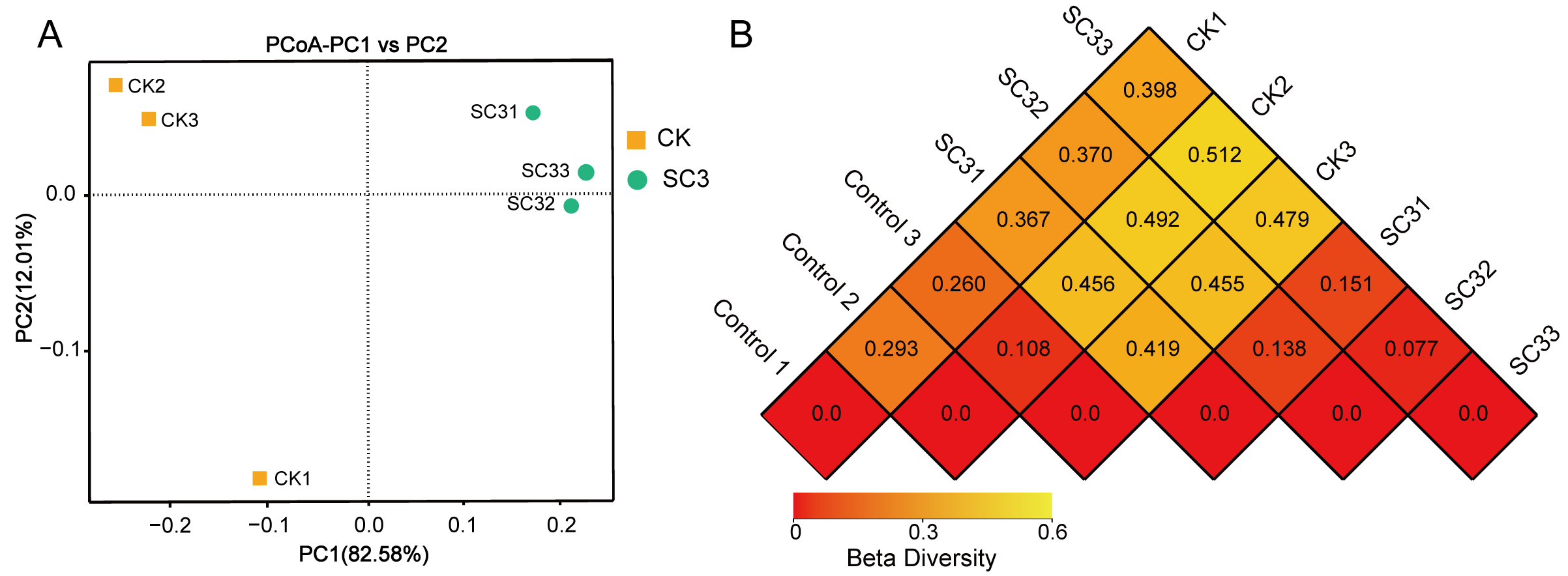

Supplement: Supplementary file 1 [file biology-12-00668-s001.zip › figure S4.tif]

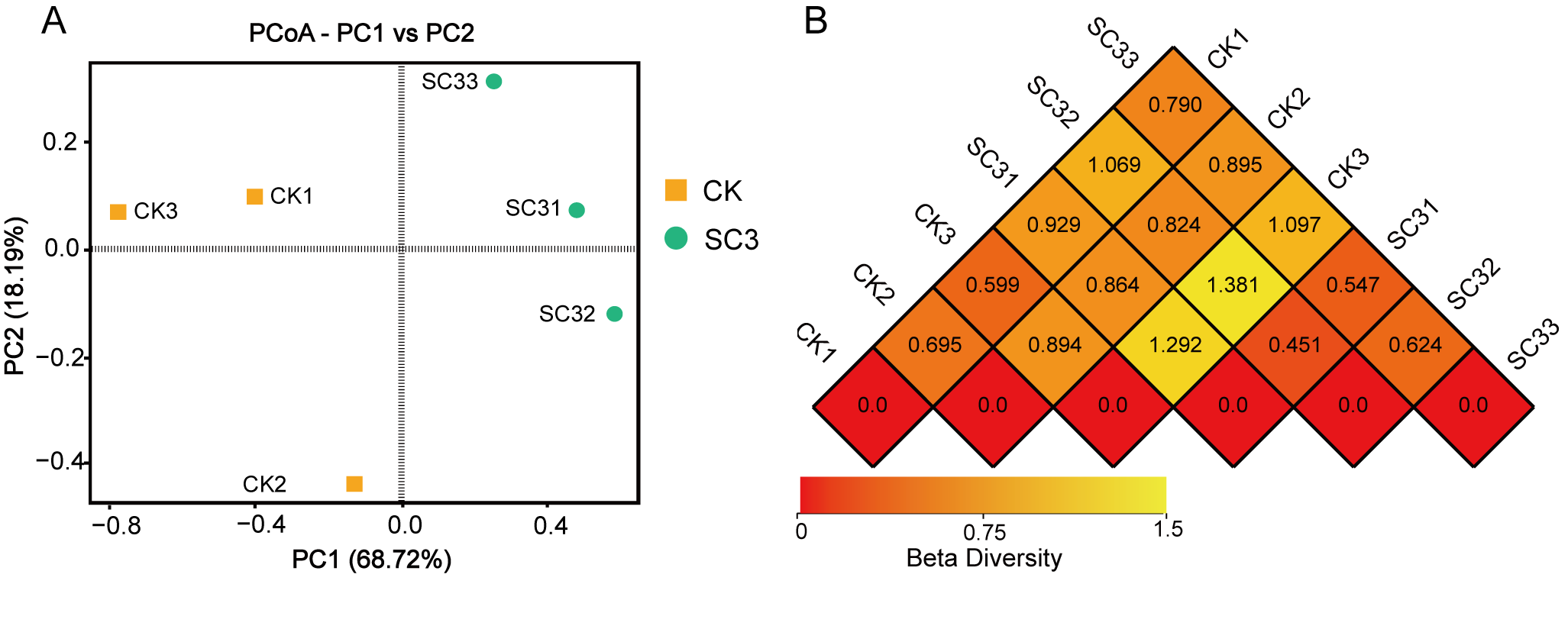

Supplement: Supplementary file 1 [file biology-12-00668-s001.zip › figure S5.tif]

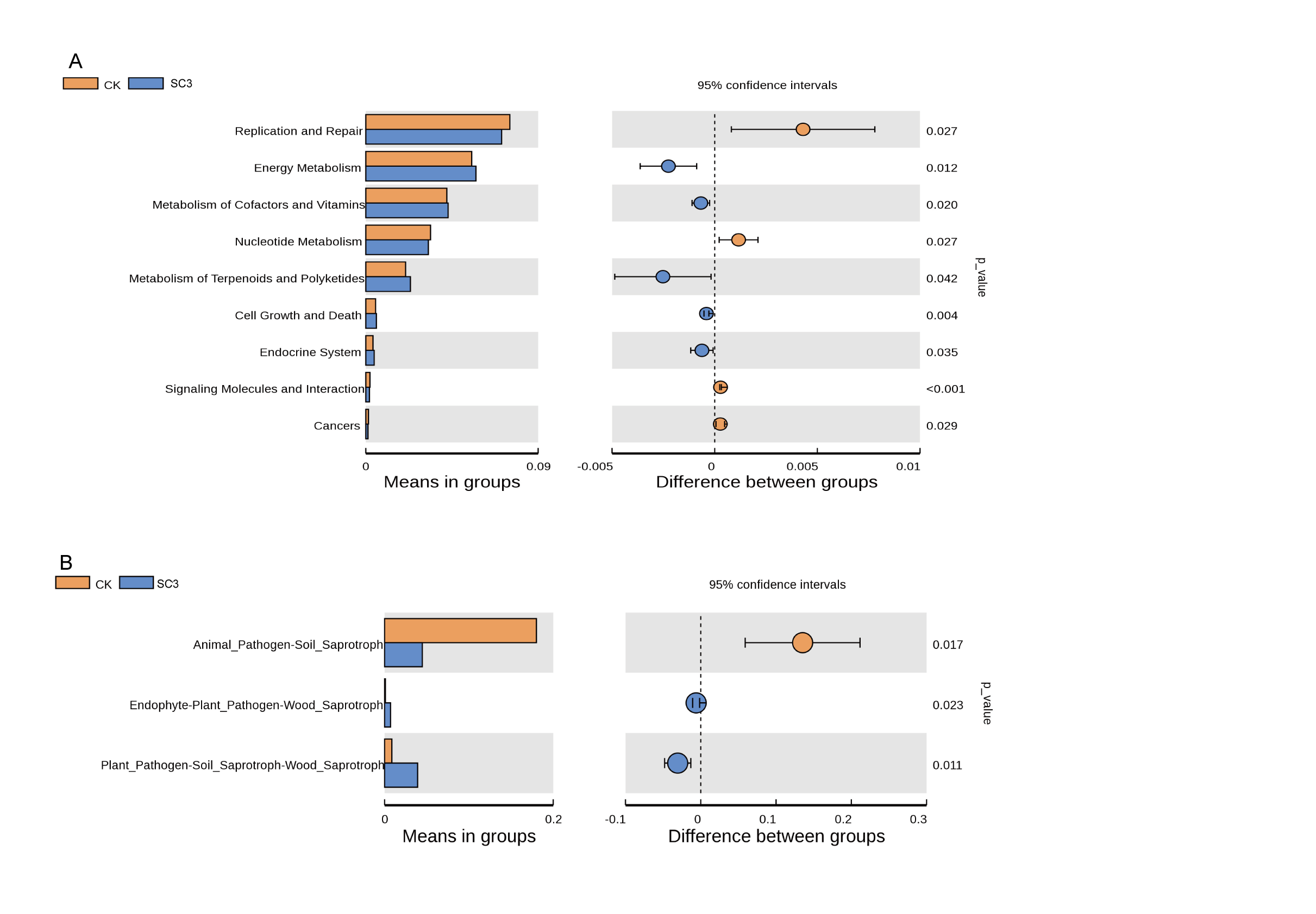

Supplement: Supplementary file 1 [file biology-12-00668-s001.zip › figure S6.tif]
